# Teaching interprofessional collaboration among future healthcare professionals

**DOI:** 10.3389/fpsyg.2023.1185730

**Published:** 2023-05-26

**Authors:** Mathias Kauff, Thorsten Bührmann, Friederike Gölz, Liane Simon, Georg Lüers, Simone van Kampen, Olaf Kraus de Camargo, Stefanus Snyman, Britta Wulfhorst

**Affiliations:** ^1^Medical School Hamburg, Hamburg, Germany; ^2^CanChild Centre for Childhood Disability Research, McMaster University, Hamilton, ON, Canada; ^3^Centre for Community Technologies, Nelson Mandela University, Gqeberha, South Africa

**Keywords:** interprofessional education, interprofessional competencies, healthcare professionals, medical education, intergroup contact, social identity

## Abstract

Healthcare has become more complex in recent years. Such complexity can best be addressed by interprofessional teams. We argue that to ensure successful communication and cooperation in interprofessional teams, it is important to establish interprofessional education in health-related study programs. More precisely, we argue that students in health-related programs need to develop interprofessional competencies and a common language, experience interprofessional contact, build inclusive identities and establish beliefs in the benefit of interprofessional diversity. We give examples how these goals can be implemented in interprofessional education. We also discuss challenges and future avenues for respective research healthcare professionals.

## 1. Introduction

Effective communication and cooperation among healthcare professionals (HCPs) is a requirement for modern and well-functioning health services (World Health Organization, 2010). We argue that interprofessional education of future HCPs can pave the way for successful interprofessional collaboration in healthcare practice.^1^ Interprofessional education (IPE) is characterized by “occasions when members or students of two or more professions learn about, with and from each other, to improve collaboration, and the quality of care and services” (Centre for the Advancement of Interprofessional Education (CAIPE), 2019).

In this article, we focus on preconditions that need to be addressed in the education of HCPs. We believe that IPE should be theory-driven and evidence-based (Michalec and Lamb, 2020). We, hence, attend to theories that call for basic measures. Building on those, we then broadly introduce practical examples of IPE. Finally, we summarize our position and discuss future challenges.^2^

## 2. Basic preconditions for interprofessional collaboration

In this section, we discuss basic conditions that relate to future HPEs’ openness for and ability to cope with interprofessional collaboration. We focus on broad measures that should be considered in study programs and that mainly build on social-psychological theories.

### 2.1. Interprofessional competencies and a common language

One obvious measure to prepare students to collaborate in interprofessional teams is fostering individual competencies relevant for managing interprofessional intergroup contexts (Frenk et al., 2022). Many scholars have identified core competencies for interprofessional practice (Thistlethwaite et al., 2014). A detailed discussion of these competencies is beyond the scope of this article. Nevertheless, we stress that besides broad and subject-specific competencies it is important to include competencies such as interprofessional values and ethics, handling of professional roles and responsibilities, interprofessional communication, abilities to work in a team and conflict behavior (World Health Organization, 2013; Claus and Wiese, 2019).

Furthermore, a prerequisite for successful cooperation among HCPs from different disciplines is the use of a common language. IPE needs to help students to develop such a language. One model that helps to prevent misunderstandings caused by unsystematic use of terms and concepts is the biopsychosocial model and the categories of the International Classification of Functioning, Disability and Health (World Health Organization, 2001; Kraus de Camargo et al., 2019; Ronen et al., 2020). This classification serves as cross-disciplinary framework and helps to describe in a standardized way an individuals’ functioning – and the impact of contextual factors on functioning – from a biopsychosocial perspective (World Health Organization, 2001).

### 2.2. Overcoming stereotypes through intergroup contact

Besides the evident fostering of interprofessional competencies and a common language, there are more basic challenges that need to be addressed. One important aspect of IPE in this regard is to provide future health professionals with opportunities to learn from each other and to overcome stereotypes.

Michalec et al. (2013) surveyed 638 students from six different health professions. The results revealed that stereotypes about the different professions varied and that perceptions of the own profession were more positive than perceptions of other professions. Moreover, Lewitt et al. (2010) showed that stereotypes between medical doctors and biomedical scientists are prevalent among undergraduate students. Likewise, Hean et al. (2006) demonstrated that students from different health and social care disciplines hold stereotypical beliefs about other health and social care professions (Cook and Stoecker, 2014). Regarding the content of the stereotypes, Hean et al. study revealed that, for example, medical doctors and midwives were perceived as more competent than podiatrists and social workers, while physiotherapists and nurses were ascribed higher practical skills than occupational therapists, doctors, audiologists, pharmacists and social workers. Focusing on the content of stereotypes in the German healthcare system, Kämmer and Ewers (2022) revealed that experienced therapists (i.e., physical, occupational or speech and language), as well as midwives and nurses perceived doctors higher on academic abilities than their own group. They also perceived doctors less practical, with poorer interpersonal and teamwork skills. Needless to say, that negative stereotypes about and devaluation of other professional groups can impair communication and collaboration in interprofessional teams (World Health Organization, 2010; Ateah et al., 2011; Darmayani et al., 2020). Hence, it is of crucial importance to overcome stereotyping and devaluation.

Intergroup contact, that is encounters with members of other social groups (outgroups), has been shown to be among the most effective measures to reduce devaluation (Allport, 1954; Pettigrew and Tropp, 2011). Getting to know outgroup members reduces anxiety, increases perspective taking and enhances knowledge about the outgroup while overcoming negative stereotypes and prejudice (Pettigrew and Tropp, 2008). Therefore, it is imperative to provide students with opportunities for intergroup contact with students from other professions, enabling mutual learning and invalidation of negative attitudes (Hean and Dickinson, 2005; Carpenter and Dickinson, 2016). In fact, a plethora of studies shows that intergroup contact between students of different health professions can increase favorable attitudes towards other professions (Carpenter, 1995; Rudd et al., 2016; Mette and Hänze, 2020). White et al. (2019), for example, demonstrated that public health education students held more positive attitudes about the academic skills of nursing students after completion of a semester-long IPE program than students in a control group that did not complete this program.

Some aspects need to be considered when using contact to reduce stereotypes and prejudice between different professions. First, intergroup contact has been shown to be especially effective when groups share similar status, work co-operatively on a task and have common goals in the contact situation (Pettigrew and Tropp, 2006). Therefore, interactions between students of different health professions should be designed to enable interprofessional collaboration, leading to the accomplishment of shared goals. Given the status-differences between different professions, as well as the strict hierarchy in many healthcare systems (Ewers and Schaeffer, 2019), it is of special importance to create interprofessional encounters in which members of different groups meet each other on eye-level.

Second, intergroup contact is also more effective when supported by persons with authority (Pettigrew and Tropp, 2006). Accordingly, educators and other individuals responsible for study programs should emphasize that intergroup contact between different professions is an important part of their agenda.

Third, support by authorities could also help dealing with a recently identified shortcoming in the intergroup contact literature. Until lately, researchers have overlooked that contact opportunities are not necessarily exploited and that some individuals actively avoid contact with members of other groups (Al Ramiah et al., 2015; McKeown and Dixon, 2017). IPE could implement programs that purposefully bring students from different professions together. Another option, however, would be to build on an individual’s contact motives and clarify that participation in programs fostering contact have a benefit for students: Students should be made aware that contact with other professional groups can satisfy their self-expansion motives, willingness to gain knowledge and aim to advance their own professional career (Paolini et al., 2016; Stürmer and Benbow, 2018).

### 2.3. Building an inclusive social identity

Intergroup contact does not only enable mutual learning and the facilitation of favorable perceptions of outgroup members, it can also help to build a shared social identity between members of different social groups (Pettigrew and Tropp, 2011). Social identity theory (Tajfel and Turner, 1979) claims that individuals’ membership in social groups are an important part of their self-concept. Belonging to social groups provides individuals with a social identity (for a recent discussion of the role of professional identities, see Greco et al., 2022). Moreover, the theory posits that individuals are motivated to achieve and maintain a positive social identity. Accordingly, groups to which individuals belong (ingroups) are evaluated more positively than outgroups. Given that the positivity of one’s social identity is always dependent on the superiority of ingroups over outgroups, it is not surprising that HCPs and students in health-related study programs tend to favor their professional group over others.

One way to overcome biases in the evaluation of the in- vs. the outgroup is to change the understanding of the structural relationship between groups. For example, by establishing a new group that includes former in- and outgroup members (Gaertner et al., 1989). Brown and Hewstone (2005) propose that animosity between groups can best be reduced by creating a joint superordinate group that includes the in- as well as outgroups. In the context of IPE that can be done by building work groups (or courses) that include various subgroups of students from different professions. In the context of these groups, students’ social identity is shaped by their identification with their profession’s group as well as the interprofessional work group (Michalec et al., 2021). On a more abstract level, universities can also be a common superordinate group. Universities should convey that they not only equip students to work in their respective healthcare professions, but that they constitute an overarching “health professions family” that is committed to educate health-professionals-in-general. Students should, hence, not only be regarded as students of psychology, medicine, nursing, or social work but – in addition – as (future) HCPs (Khalili et al., 2013; Joynes, 2018).

However, a hierarchical structure in which different professions are nested in a joint superordinate profession, may also lead to a devaluation of certain professions. This may be due to a lack of prototypicality of these groups for the superordinate group (Mummendey and Wenzel, 1999; Reese et al., 2016). When two groups are part of a superordinate group, it may be the case that members of one group perceive their ingroup but not the outgroup as prototypical for the overarching group. This may lead to a devaluation of the outgroup (Wenzel et al., 2007). It could, for example, be that medical students believe that they are part of a larger group that also includes nursing students. However, the medical students perceive themselves as more prototypical for the larger group “health experts” and, consequently, devalue nursing students (for a similar effect among primary-school teachers vs. high-school teachers, see Waldzus et al., 2004). One antidote for this process can be found in the characterization of the superordinate group. Waldzus et al. (2003) showed that a definition of the superordinate as diverse can reduce perceptions of higher relative and decrease devaluation of subordinate outgroups. Hence, it is not only important to introduce a superordinate group as outlined above, but to also establish a self-image within this group which is determined by the group’s diversity. Universities should enable students to identify with a larger encompassing “health-professions-in-general” group at the respective institution. In addition, universities need to construct this larger group in a way that it is defined by its diversity. Accordingly, interprofessionalism should be an important part of a university’s mission statement.

### 2.4. Believing in diversity

“Diversity refers to differences between individuals on any attribute that may lead to the perception that another person is different from self” (Van Knippenberg et al., 2004, p. 1008), Individuals’ professional background constitutes one dimension of diversity. A plethora of research tackled the question whether diverse groups outperform homogenous groups when it comes to group functioning and productivity (Meyer, 2017). We now know that the relationship between diversity and outcomes of workgroups is dependent on a number of moderating variables (Van Knippenberg et al., 2004) – among them diversity-beliefs (van Knippenberg and Haslam, 2003; Homan et al., 2019). Diversity beliefs can be defined as “beliefs individuals hold about how group composition affects group functioning, i.e., whether individuals perceive diversity as beneficial, detrimental or neutral for the group functioning” (van Dick et al., 2008, p. 1467). Studies have demonstrated that within diverse groups it is crucial that group members hold pro-diversity beliefs (i.e., beliefs that diversity is an asset to the group). Van Dick et al. (2008), for example, showed that members holding pro-diversity beliefs were more strongly identified with diverse groups than those that held a critical stance on diversity. Furthermore, Homan et al. (2008) demonstrated that diverse groups were more productive than homogenous groups when group members held beliefs in the instrumentality of diversity (for an overview, see Leslie and Flynn, 2022).

To summarize, diverse groups (among them groups that consist of members with different educational/professional backgrounds) can outperform homogenous groups when members believe in the benefit of diversity for group functioning. Accordingly, health professions educators should not only stress the existence of diversity as a value of a superordinate group, but emphasize that diversity makes the group more productive and better placed to solve complex health problems. As a consequence, identification with, information elaboration within and performance of the group should increase. In the context of IPE, the benfit of diversity can be stressed by the application of the biopsychosocial model, which implies that illness and health are the result of an interaction between biological, psychological and social factors (World Health Organization, 2001). Health-related issues can best be addressed by practitioners from different professions collaborating interprofessionally.

Pro-diversity beliefs can also be used to reduce prejudice and mutual discrimination (Kauff et al., 2021). Conflict between professions often results from different forms of intergroup threat (Stephan et al., 2016). Nursing students, for example, might feel threatened by medical students because they fear that medical students are allocated more resources. Students of social work might feel threatened by the idea that psychology students are perceived as more competent in counseling work in clinical settings. Kauff and Wagner (2012) could show that pro-diversity beliefs can reduce such perceptions of threat and, consequently, reduce conflict between groups.

## 3. Teaching interprofessionalism in action

Successful implementation of IPE requires measures on various levels, such as institutional commitment, social interactions between students and the integration of IPE in all health professions curricula, including a uniform way of assessing interprofessional competencies (AIPHE, 2014). As example, we will elaborate on problem-based learning (PBL) as general approach to foster interprofessional competencies. We also provide a short overview of a concrete example of an interprofessional module.

### 3.1. Problem-based learning

From an action-theoretical perspective, the acquisition of interprofessional competencies cannot be taught directly. However, learning environments and opportunities can promote the acquisition of such competencies. PBL meets these criteria and is often used in the context of IPE (Aldriwesh et al., 2022). PBL can be conceived as a higher-level learning approach (Servant-Miklos, 2020) characterized by consistent case orientation and interactive group-learning. PBL build on clearly defined procedures and division of roles (Barrows, 1996; Moust et al., 2005). PBL can be combined with interprofessional learning in a low-threshold way, either as a curriculum-integrated format or in a cross-curricular event format.

We have experience with the latter format. Once a semester, students from up to eight different study programs are invited to work for one day on complex case examples in interprofessional groups. The cases focus on patient problems (e.g., a neglected child with multiple diagnoses, fails at school), group topics (e.g., dealing with shame in an interprofessional team), or institutional concerns (e.g., designing a dementia-sensitive hospital). The students are accompanied by trained tutors and present their interprofessional solutions in short presentations to a large plenum.

PBL fosters interaction between members of different professions. This interaction includes an expression of views from different health professions during the negotiation of phenomena and problems, the joint construction of knowledge, fostering a common identity and individuals’ the metacognitive act as well as group reflection. It has been shown that PBL interventions promote communication-related competencies and improve mutual attitudes of members of different professions (Goelen et al., 2006; Dahlgreen, 2009; Lin et al., 2013; Braßler and Dettmers, 2016).

### 3.2. Module “digital health”

We also implemented an interprofessional elective module in which students are faced with real problems from healthcare practice that often require digital solutions (e.g., a smartphone application for health behavior). Students are brought together to collaborate interprofessionally face-to-face. The interaction is supported by an interprofessional team of lecturers. The enrolment in the module presents a benefit for the students as it deals with a timely and relevant topic that are not encountered in other courses. Ideally, students become aware of differences between professions and how collaboration can lead to better person-centered solutions. They also learn that solution-oriented interprofessional project work can be transferred directly into practice. This module fosters interprofessional contact between students, helping them to build an overarching superordinate identity that embraces diversity. Moreover, students experience the benefit of interprofessional diversity.

## 4. Discussion

Challenges within health care systems have drastically changed over the last decades as patient care became more complex (Frenk et al., 2022). Likewise, the distribution of tasks between HCPs has changed (Hahn, 2011). To ensure successful communication and cooperation in health teams, future HCPs need to be prepared for interprofessional collaborative practice. In this article, we argue that IPE in health professions education needs to (a) convey interprofessional competencies and a common language, (b) provide opportunities for intergroup contact, (c) develop a common identity and (d) facilitate beliefs in the benefit of diversity. This can be achieved through problem-based learning and in modules that bring together students from different professions to collaborate on relevant societal issues.

From our own experience, we know that implementing IPE can be difficult (Helms and Held, 2020). Often students are caught up in their profession’s identity, in professional tribalism and in established hierarchies between professions. Moreover, guidelines for examinations, strict regulations for study courses and a lack of resources make it difficult to implement IPE (Ghebrehiwet et al., 2016; Tong et al., 2016; Busari et al., 2017; Hämel and Vössing, 2017; Findyartini et al., 2019). Frenk et al. (2022) discuss new challenges regarding the implementation of IPE in more detail (see also Wetzlmair et al., 2021; for examples of implementation of IPE during the pandemic, see Alrasheed et al., 2021; Engelmann et al., 2021). Many of these are direct consequences of the COVID-19 pandemic. For example, face-to-face learning in classrooms has declined, which complicates collaborative learning even more. At the same time, the pandemic increased demand for complex health services and, consequently, interprofessional collaboration.

Studies investigating the effectiveness of IPE address different outcomes related to performance (Langlois, 2016; Champagne-Langabeer et al., 2019; Au, 2022). Future work needs to address how we can best evaluate the success of IPE (Kahaleh et al., 2015; Anderson et al., 2016). Robust evidence how IPE contributes to successful cooperation and communication and how it reflects on the aforementioned broader prerequisites is scarce. We trust this article helps to stimulate respective work.

## Data availability statement

The original contributions presented in the study are included in the article/supplementary material, further inquiries can be directed to the corresponding author/s.

## Author contributions

MK wrote the first draft of the manuscript. SK and BW wrote sections of the manuscript. All authors contributed to the article and approved the submitted version.

## Conflict of interest

The authors declare that the research was conducted in the absence of any commercial or financial relationships that could be construed as a potential conflict of interest.

## Publisher’s note

All claims expressed in this article are solely those of the authors and do not necessarily represent those of their affiliated organizations, or those of the publisher, the editors and the reviewers. Any product that may be evaluated in this article, or claim that may be made by its manufacturer, is not guaranteed or endorsed by the publisher.

## Footnotes

^1^We define interprofessional collaboration as a co-operation that is characterized by interdependence, shared responsibilities within a team with common goals, joint commitment, and mutual respect (World Health Organization, 2010; Khalili et al., 2019). basic preconditions for interprofessional collaboration that needs to be addressed in health-care education.

^2^We would like to direct the readers’ attention to the fact that the present article itself is a result of an interprofessional collaboration. The contributing authors’ scholarly background encompasses a variety of disciplines such as pedagogics, medicine, psychology, arts, and social work.
